# Use of platelet-rich fibrin in fat grafts during facial lipostructure

**DOI:** 10.3389/fsurg.2022.923342

**Published:** 2022-10-27

**Authors:** Zhaoxiang Zhang, Lihong Qiu, Dong Cui, Jian Geng, Chenggang Yi

**Affiliations:** Department of Plastic Surgery, Xijing Hospital, Fourth Military Medical University, Xi’an, China

**Keywords:** platelet-rich fibrin (PRF), fat graft, facial lipostructure, facial lipofilling, plastic surgery

## Abstract

**Background:**

This review was designed to discuss the safety and efficacy of using platelet-rich fibrin (PRF) in fat grafts during facial lipostructure.

**Methods:**

From January 2018 to December 2021, 650 fat grafts for facial lipostructure were performed in the authors' department. According to their wishes, we divided the patients into two groups: 498 patients were treated with autologous fat injection (control group), and 152 patients were treated with autologous fat injection combined with PRF. All of the patients were monitored for at least six months. The effects were evaluated *via* physician assessment and patient satisfaction rates, and the incidences of complications were compared.

**Results:**

All the cases had a degree of improvement after treatment. The patient satisfaction rate was 55.3% in the PRF group and 43.4% in the control group. In all, 68.4% of the patients in the PRF group and 58.2% in the control group indicated that one-stage surgery was sufficient to achieve the desired effect. According to the evaluation conducted by the plastic surgeon, 59.2% of patients in the PRF group and 47.0% in the control group achieved a perfect effect. A total of 76.3% of patients in the PRF group and 63.9% in the control group reported that one surgery achieved satisfactory results. The difference between the PRF and control groups was statistically significant.

**Conclusion:**

Using an autologous fat graft during facial lipostructure is beneficial and safe when combined with PRF. The combination may enhance the effect and satisfaction rate. Further research and prospective clinical studies are needed to understand the role of PRF in fat grafting.

## Introduction

When people are young, facial fat is diffuse, plentiful, and balanced, making the face look perfect, but as the face ages, the fat distribution becomes uneven. The fat in some compartments will be deposited, or hypertrophy will occur; other fat compartments will remain atrophic (1). The transfer of fillers into atrophic compartments can restore a sense of balance and youth. Autologous fat is the ideal filler for patients who want to add contour and projection to the aging face because fat is versatile, enduring, safe, and economical. Therefore facial lipostructure surgery has become increasingly popular. However, avoiding fat absorption is still a challenge for plastic surgeons. Many techniques described in the literature are used to improve the maintenance of volume; the most interesting techniques in the practical application of fat grafting are the use of prosurvival strategies, such as platelet-rich plasma (PRP) (2–7) and platelet-rich fibrin (PRF) (8–10). This study was designed to assess the efficacy of PRF combined with a fat graft during facial lipostructure surgery.

## Patients and methods

This retrospective study involved patients who received fat grafts for facial lipostructure from January 2018 to December 2021 in the author's department. Those excluded from this study included patients who underwent unilateral facial lipostructure surgery, those who had fat grafts previously during facial lipostructure, who underwent additional procedures such as facelift or blepharoplasty at the same time, who could not be followed up for six months, and those whose body mass index (BMI) was not 18.5–22.9. A total of 650 patients who underwent lipostructure were included in this study. Based on patient preference, 152 patients (2 men and 150 women) aged 21 to 42 years with a mean age of 28 ± 6.5 years underwent bilateral facial lipostructure surgery with PRF, and 498 patients (16 men and 482 women) aged 20 to 45 years with a mean age of 27 ± 6.9 years were treated with common fat grafts alone as the control group (Table 1).

**Table 1:** Patients information.

Group	*n*	Male	Female	Mean age	BMI
PRF + fat graft (PRF group)	152	2	150	28 ± 6.5	18.5–22.9
Fat graft (control group)	498	16	482	27 ± 6.9	18.5–22.9

Note: PRF, platelet-rich fibrin.

All patients were examined preoperatively to estimate the total amount of fat to be transplanted into each area of the face, and those areas were marked. All patients received general anesthesia for the operation, and the same surgeon performed all steps of fat harvesting, processing, and injection according to the protocols described by Coleman (11). The study protocol complied with the Declaration of Helsinki and was approved by the Xijing Hospital Ethics Committee (no: LL-KY-20131226). A signed written informed consent was obtained from each of the enrolled patients.

## Fat harvesting and processing

There have been no publications indicating increased viability from any one donor site (12, 13); therefore, the donor sites were chosen based on convenient access and abundant amounts of adipose tissue. The abdomen or thigh areas usually represent ideal donor sites. A 3 mm incision was made with a number 11 scalpel blade, and the anesthetic solution (500 ml saline solution, 20 ml of 20 mg/ml lidocaine, and 0.5 ml of 1 mg/ml epinephrine) was injected into the donor areas with a blunt cannula. Then, the adipose tissue was harvested through the same incision with a two-hole Coleman harvesting blunt cannula (2 mm in diameter, 15 to 23 cm in length), and the cannula was attached to a 10 ml Luer-Lok syringe whose plunger was pulled back to the 1 ml marker to maintain a gentle negative pressure. When filled, the syringe was placed in a centrifuge with resterilized containers, and the centrifugation speed was 3000 rpm (approximately 800 g) for three minutes. After centrifugation, the contents of the syringe were divided into three layers. The upper layer consisted primarily of oil, which had the lowest density. This oil layer was usually decanted. The lowest layer contained primarily blood, water, and any aqueous element eliminated when the syringe was withdrawn. The middle layer was primarily fatty tissue that would be grafted. For the control group, the refined fat was then transferred to 1 ml Luer-Lok syringes for injection.

## Platelet-rich fibrin preparation

**Figure 1:**
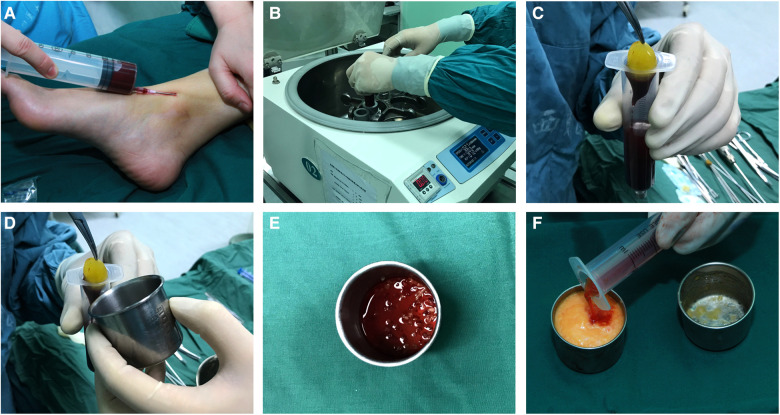
Platelet-rich fibrin preparation: (**A**) intravenous blood was collected with a disposable syringe; (**B**) the blood was centrifuged at 3000 rpm for 10 min; (**C**) a platelet-rich fibrin clot was taken out of the syringe; (**D**) platelet-rich fibrin clot; (**E**) the platelet-rich fibrin clot was cut in fragments of 1 to 2 mm; (**F**) the fragments of the PRF clot were mixed with the refined fat, and the mixture was then transferred to 1 ml luer-Lok syringes for injection.

Then, 20–60 ml of intravenous blood (the blood volume was consistent with the amount of fat to be injected) was collected with a disposable syringe to obtain the PRF and was immediately transferred to disposable tubes and centrifuged (Centrifuge 5084 R; Eppendorf, Germany) at 3000 rpm (approximately 800 g) for 10 min in the centrifuge according to the protocol described by Choukroun et al. (14–16). The speed of blood collection and transfer to the centrifuge was the key to obtaining a clinically usable PRF clot because no anticoagulant was added to the blood. After centrifugation, the contents of the tubes were divided into three layers based on differential densities: a base of red blood corpuscle at the bottom, acellular plasma on the surface, and finally, a clot of fibrin PRF between the two. The PRF clot was taken out and placed into a sterile dish, and then it was cut into fragments of 1 to 2 mm with microsurgical scissors and tweezers. For the PRF group, the fragments were mixed with the refined fat at a volume ratio of 1:2, forceps were used for repeated stirring, then gently inverted three to four times to allow a better mixture (3, 17, 18), and the mixture was then transferred to 1 ml Luer-Lok syringes for injection (Figure 1).

## Fat injection

Our usual injection areas were the following: forehead and temple regions, nasojugal fold, cheek, lips, chin, marionette lines, and nasolabial folds, and all the areas were bilateral. Incision sites were placed at frequent intervals along the hairline or other hidden parts through the 2 mm incision. The anesthetic solution was injected into the recipient area to inhibit bruising and prevent vascular cannulization. Fat was always transferred with a blunt 18 or 17 gauge cannula attached to the fat-filled syringe. As the cannula was withdrawn, the fatty tissue was injected into the appropriate plane; 0.1 ml or less of fat was deposited in each tunnel. It was critical to make sure all layers were grafted and that the grafting was started at the deepest point. The incisions were closed with 7–0 nylon sutures.

## Results evaluation

Plain photographs were obtained preoperatively and systematically at six months postoperatively, and all photographs were taken under the same conditions. Two approaches were used to assess the results of the operation. The first was a patient satisfaction assessment acquired from them through a questionnaire after a six-month follow-up. The contents of the questionnaire included the following: the overall patient satisfaction with the operation (possible answers: satisfied, acceptable, dissatisfied), whether one-stage surgery was enough to achieve the desired effect, and if there was a need for a staged operation (yes, no) (Table 2). The second approach was a questionnaire filled out by a plastic surgeon who did not perform the procedure. The evaluation was conducted with photos of the patient to compare the images before surgery and six months after surgery. The contents of the questionnaire included the following: the general effect of the operation (possible answers: good, sufficient, bad) and whether one-stage surgery could achieve the therapeutic effect (yes, no) (Table 3). The plastic surgeon who evaluated the operation results was blinded, but the surgeon who performed the surgery and the patients were not.

**Table 2:** Patients possible answers included in the questionnaire.

Question	Possible answers	PRF group, *n* (%)	Control group, *n* (%)	*p*-value
1. Overall satisfaction	Satisfied	84 (55.3)	216 (43.4)	0.031
	Acceptable	39 (25.6)	150 (30.1)	
	Dissatisfied	29 (19.1)	132 (26.5)	
2. One stage surgery	Yes	104 (68.4)	290 (58.2)	
	No	48 (31.6)	208 (41.8)	0.024

Note: PRF, platelet-rich fibrin; *p*-value was calculated by Chi-square test.

**Table 3:** Surgeon possible answers included in the questionnaire.

Question	Possible answers	PRF group, *n* (%)	Control group, *n* (%)	*p*-value
1. Surgery effect	Good	90 (59.2)	234 (47.0)	0.028
	Sufficient	38 (25.0)	153 (30.7)	
	Bad	24 (15.8)	111 (22.3)	
2. One stage surgery	Yes	116 (76.3)	318 (63.9)	0.004
	No	36 (23.7)	180 (36.1)	

Note: PRF, platelet-rich fibrin; *p*-value was calculated by Chi-square test.

## Statistical analysis

Statistical analyses included the chi-square test and were performed with SPSS software (version 18.0; SPSS Inc., Chicago, IL, USA). A *p*-value of 0.05 or less was considered statistically significant.

## Results

**Figure 2:**
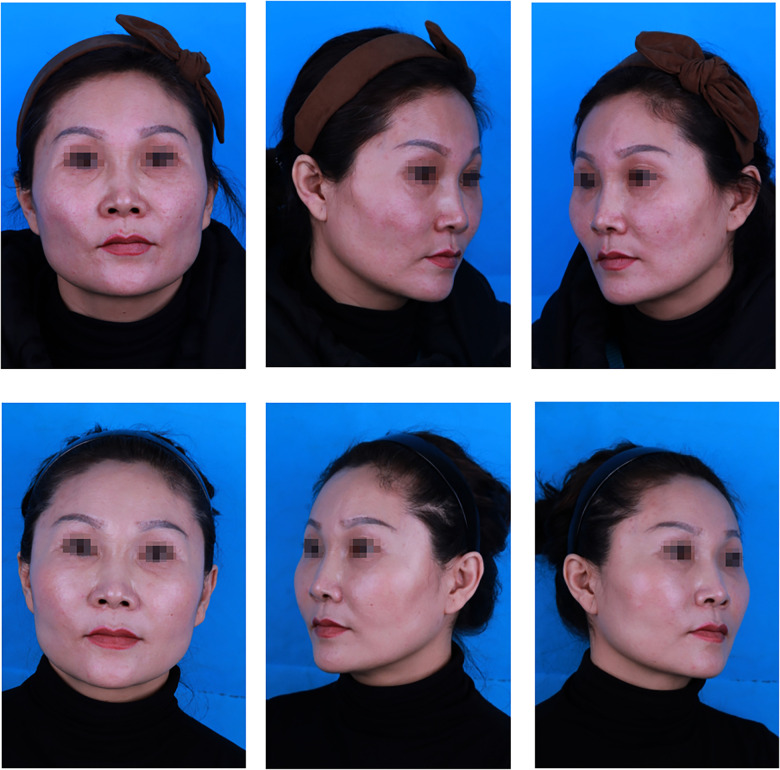
A 43-year-old patient: (top) preoperative view; (bottom) postoperative view 12 months after fat graft combined with PRF to the cheek, nasojugal folds and temporal regions, and 12, 5, and 24 ml of PRF-assisted fat were lipotransferred into each region (bilateral), respectively.

**Figure 3:**
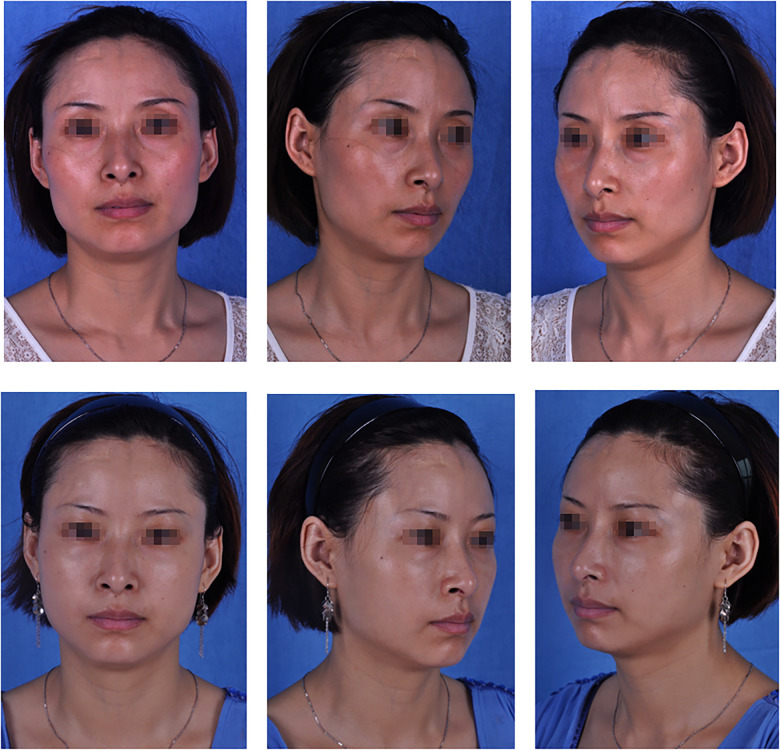
A 36-year-old patient: (top) preoperative view; (bottom) postoperative view 6 months after fat graft combined with PRF to the upper and lower eyelids, cheek, nasolabial folds and temporal regions, and 4, 3, 6, 4 and 20 ml of PRF-assisted fat were lipotransferred into each region (bilateral), respectively.

The results of our follow-up evaluation are summarized in Tables 2, 3. According to the patient answers, the overall patient satisfaction rate was 55.3% in the PRF group and 43.4% in the control group. A total of 68.4% of patients in the PRF group and 58.2% in the control group indicated that one-stage surgery was sufficient to achieve the desired effect. There was a significant difference between the PRF and control groups (*p* < 0.05 for both) (Table 2). According to the evaluations conducted by the plastic surgeon, 59.2% of patients in the PRF group achieved a good effect, which was significantly higher than in the control group (47.0%). A total of 76.3% of patients in the PRF group and 63.9% in the control group reported that one surgery achieved satisfactory results. Similarly, the difference between the PRF and control groups was statistically significant (*p* < 0.05 for both) (Table 3). The results of the PRF group are shown in Figures 2, 3.

Our study showed no fat embolism, vascular/nervous injury, infection, necrosis, massive edema, prolonged bruising, or severe pain. Six patients in the PRF group and 13 in the control group had a slight hematoma postoperatively; all of these patients recovered after two weeks. Under-correction was the most frequent complication in both groups.

## Discussion

Autologous fat grafting was first reported in 1893 by Neuber (19). It is now one of the most common and widely performed procedures in plastic surgery. An increasing number of surgeons consider fat as the closest thing to an ideal facial filler because (20) adipose tissue is readily available, safe, inexpensive, and simple to obtain. Because the fat tissue used in these procedures is autologous, there is no risk of host rejection or other immunologic responses. As a facial filler, it is soft and natural, and it improves the skin's quality at no additional cost to patients (21). However, grafted fat can be absorbed into the body, and the resorption rate is usually high and difficult to control (22). Much research is being done to search for ways to refine and improve our results, but a standardized method to improve the survival rate of fat grafts has not yet been adopted by all practitioners. There is no agreement on the ideal method for grafting fat to achieve the best results (23).

Many studies have confirmed that the Coleman method is the currently recognized fat harvesting, processing, and injection method (21). The anesthetic solution was injected into the donor area with a blunt cannula. Then the adipose tissue was harvested through the same incision with a two-hole Coleman harvesting blunt cannula. The cannula was attached to a 10 ml Luer-Lok syringe, and the plunger was pulled back to the 1 ml marker to maintain a gentle negative pressure. When filled, the syringe was placed into a centrifuge and centrifuged at 3,000 rpm for three minutes. After centrifugation, the middle layer was primarily fatty tissue which would be transferred to 1 ml Luer-Lok syringes for injection through a 2 mm incision. The anesthetic solution was injected into the recipient area to inhibit bruising and prevent vascular cannulization. The fat was transferred with a blunt cannula attached to the fat-filled syringe. As the cannula was withdrawn, the fatty tissue was injected into the appropriate plane. With each tunnel, 0.1 ml or less of fat was deposited. It was essential to make sure that the fat distribution was uniform. This technique is the most common method of autologous fat grafting and is now being accepted by more plastic surgeons (24). Despite using this method, the absorption rate of fat is still very high; to improve the survival rate of fat grafts, some strategies have been proposed, such as tissue engineering approaches and chemical cell-stimulating factors (25–28). Currently, autologous PRP or PRF mixed with fat grafting attracts many plastic surgeons.

The PRP can stimulate the regeneration and repair of different tissues *via* the activation and secretion of many growth factors and other cytokines stored in their alpha granules. The growth factors and cytokines can stimulate cell proliferation, chemotaxis, angiogenesis, cell differentiation, the fibrogenesis activity of fibroblasts, and extracellular matrix synthesis (29–31). The PRP was widely used in periodontal and oral surgery, spinal fusion, and cardiac bypass surgery, and successful clinical results were achieved (32–35). In the same way, PRP theoretically has the potential to enhance the survival of grafted fat, and the combination of fat grafts and PRP has been evaluated by some animal and clinical studies. In the animal studies, most research teams found that the fat graft volume and weight were higher in the PRP group than in the control group, and histopathological investigations revealed fewer cysts and vacuoles and less fibrosis in the PRP group than in the control group (7, 36–39). Only one animal study did not record any effect on fat survival after enrichment with PRP; the difference between this study and others was that the activation method to induce platelet activation was not used (40). There are also variable results in the clinical studies; many authors demonstrated that the use of PRP mixed with fat resulted in an increase in fat graft survival and function (5, 6, 9, 41–44). However, Salgarello et al. (3) and Fontdevila et al. (2) found no significant differences in the fat necrosis rate in their studies. The main reason for the two opposite results is that different PRP concentrations were used. The concentration that appears to obtain the most favorable results is 0.4 to 0.5 ml of PRP per milliliter of fat (1:2 ratio). Therefore, most current data indicate that PRP may have a dose-dependent positive effect on fat grafts (4, 45).

One of the main limitations of PRP is that most of the presynthesized growth factors are secreted within the first hour, and the additional synthesis of growth factors then continues until day eight (28, 30, 46). Furthermore, the PRP production process is complex, and anticoagulants are needed during it. Therefore, the second-generation platelet concentrate (PRF) was developed in France by Choukroun et al. in 2001 (14, 15). Some studies have shown that platelet-rich fibrin (PRF) increases vascularization by stimulating the production of angiogenesis-related factors (e.g., VEGF, basic fibroblast growth factor, plate- let-derived growth factor, and epidermal growth factor), which leads to new blood vessel formation by modulating the activation, proliferation, and migration of endothelial cells (17, 47, 48).

As a new generation of platelet concentrate, PRF has several additional advantages over traditional PRP, such as its ease of preparation and the lack of a need for any biochemical modification (46). It is the simplest and the least expensive protocol that has been developed so far. In contrast to PRP, several studies have confirmed that PRF gradually releases platelet-derived growth factor and transforming growth factor for 28 days (9, 49, 50). Moreover, the leucocytes and platelets trapped in the fibrin matrix can continue to produce high quantities of cytokines, which could intervene in the regulation of inflammatory reactions. Hence, the anti-infection ability could be enhanced (51). Unlike the PRP, PRF does not dissolve quickly after application. The strong fibrin matrix is slowly remodeled similarly as a natural blood clot, the platelets and released growth factors are combined in a chemical bond, and by slow release, the action time of the growth factors is prolonged, which will improve the survival rate of the fat (10).

Therefore, PRF seems to be more suitable than PRP for widespread use in the clinic, and some applications of this autologous biomaterial have been demonstrated in oral, maxillofacial, and plastic surgeries (14, 15, 52). In some studies on fat grafting during facial lipostructure, significant differences were found in the fat survival rates between the PRF and control groups (53–56). In our study, we tried to confirm the finding that grafts with adipose tissue mixed with PRF were better than those without it. In all, 650 patients who underwent lipostructure were included in our study. A total of 152 patients underwent bilateral facial lipostructure surgery with PRF, and 498 patients were treated with a common fat graft. Through the investigation of patients and plastic surgeons to compare the differences between the two groups, the difference between the PRF and control groups was statistically significant. However, our study was limited by the subjective nature of the evaluation carried out by the patients or surgeons; the results may be significantly different if the surgeons or patients change. Therefore, an objective and quantitative method of measuring the fat amount would be better. In conclusion, additional studies investigating the effect of using PRF in fat grafting should be developed in the future.

## Conclusion

Autologous fat grafting is still a good choice in facial aesthetic surgery, as it is a safe, effective, and durable treatment. This study found significant differences in the fat volume gain between the PRF and control groups, measured with pre- and postoperative photographs. However, our study lacks objective data to support these results and this is a limitation of the study. Further research and prospective clinical studies are needed to understand the role of PRF in fat grafting, and we believe that PRF will be used more broadly in the future.

## Data Availability

The original contributions presented in the study are included in the article/Supplementary Material, further inquiries can be directed to the corresponding author/s.
